# Supplementary material for: Characterizing the dynamics underlying global spread of epidemics
Source: Nat Commun. 2018 Jan 15;9:218. doi: 10.1038/s41467-017-02344-z (PMC5768765; doi:10.1038/s41467-017-02344-z)
Supplement: Supplementary file 1 — Supplementary Information [file 41467_2017_2344_MOESM1_ESM.pdf]

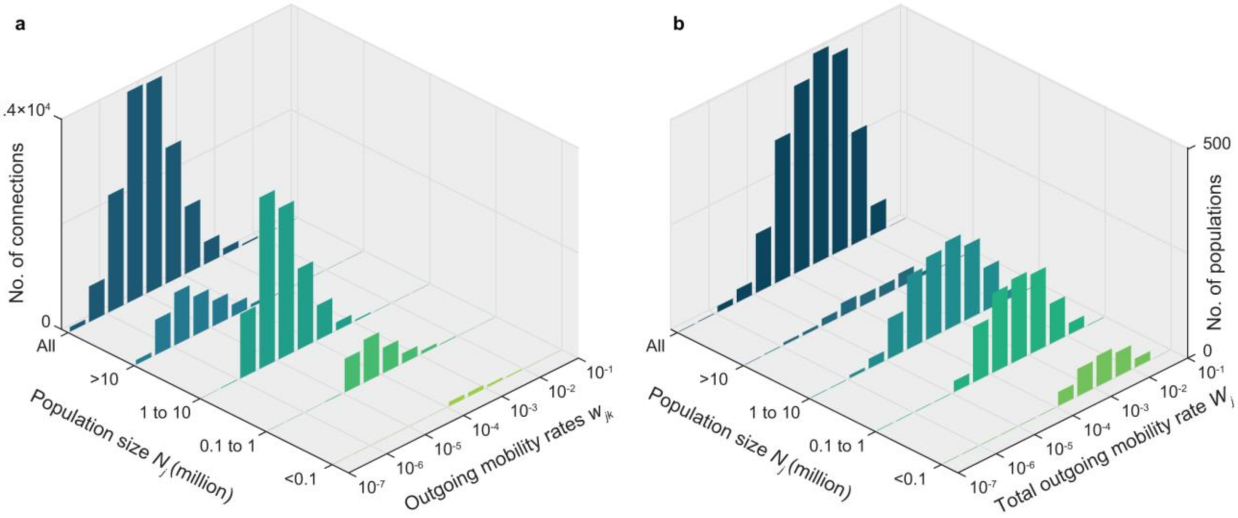

Supplementary Figure 1. Mobility rates in the WAN. a, Histograms of outbound mobility rates w_{jk} in the WAN stratified by population size. **b**, Histograms of total outbound mobility rates $W_j = \sum_k w_{jk}$ in the WAN stratified by population size.

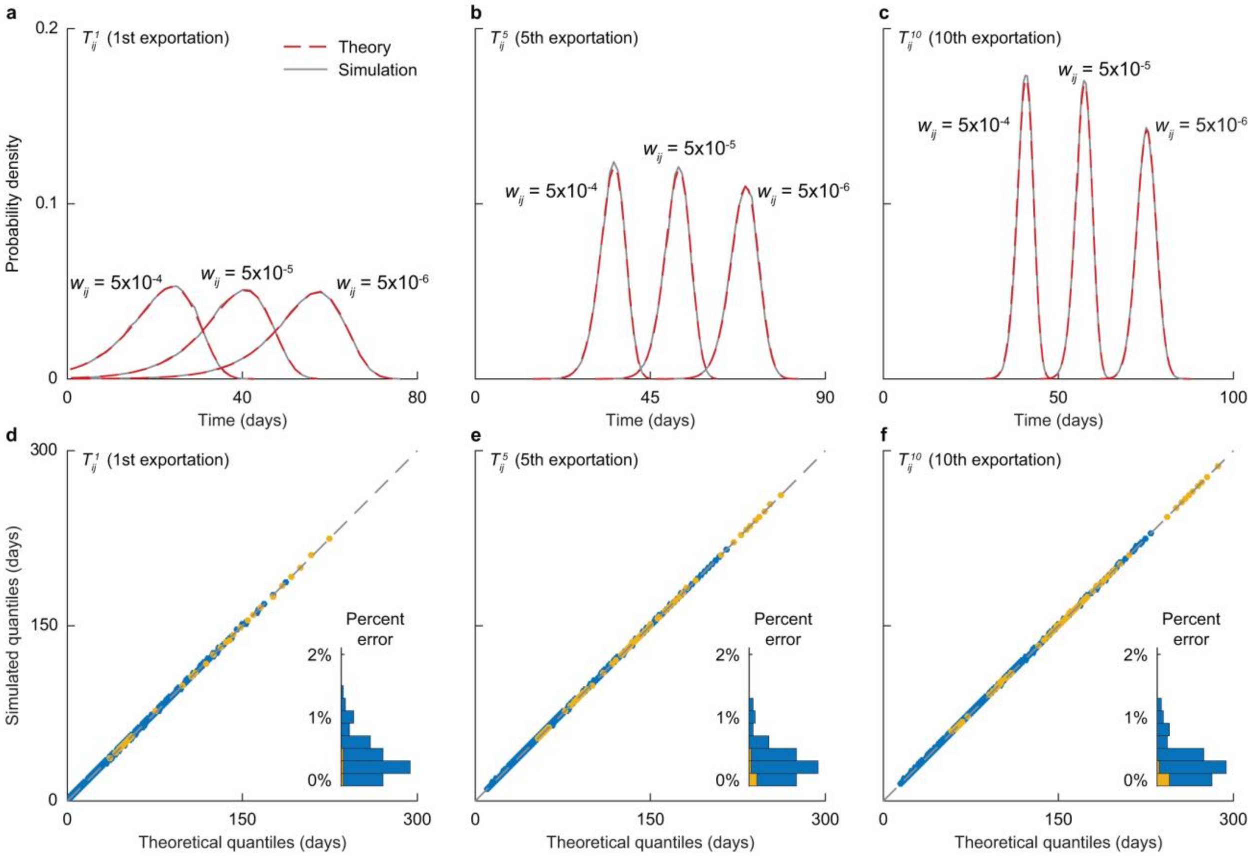

Supplementary Figure 2. Validating Assumption 1 in the two-population model. This figure is analogous to Fig. 1 but without the imposition of Assumption 2. That is, the analytical pdf of T_{ij}^{n} here is given by equation (S1). **a–c**, The analytical (red dashed lines) and simulated (grey lines) pdf of T_{ij}^{1}, T_{ij}^{5} and T_{ij}^{10} for the same scenario of exemplary influenza pandemic as in Fig. 1**a**-**c**. **d–f**, Q–Q plots for the analytical and simulated quantiles of T_{ij}^{1}, T_{ij}^{5} and T_{ij}^{10} across the same 100 scenarios considered in Fig. 1**d**-**f**. In the Q-Q plots, deviations from the diagonal indicate discrepancies between the analytical and simulated quantiles. Q-Q plots are colored in blue if the number of exportations is n or above with probability 1 and yellow otherwise. Insets show the corresponding histograms of percent error in $E[T_{ij}^{n}]$.

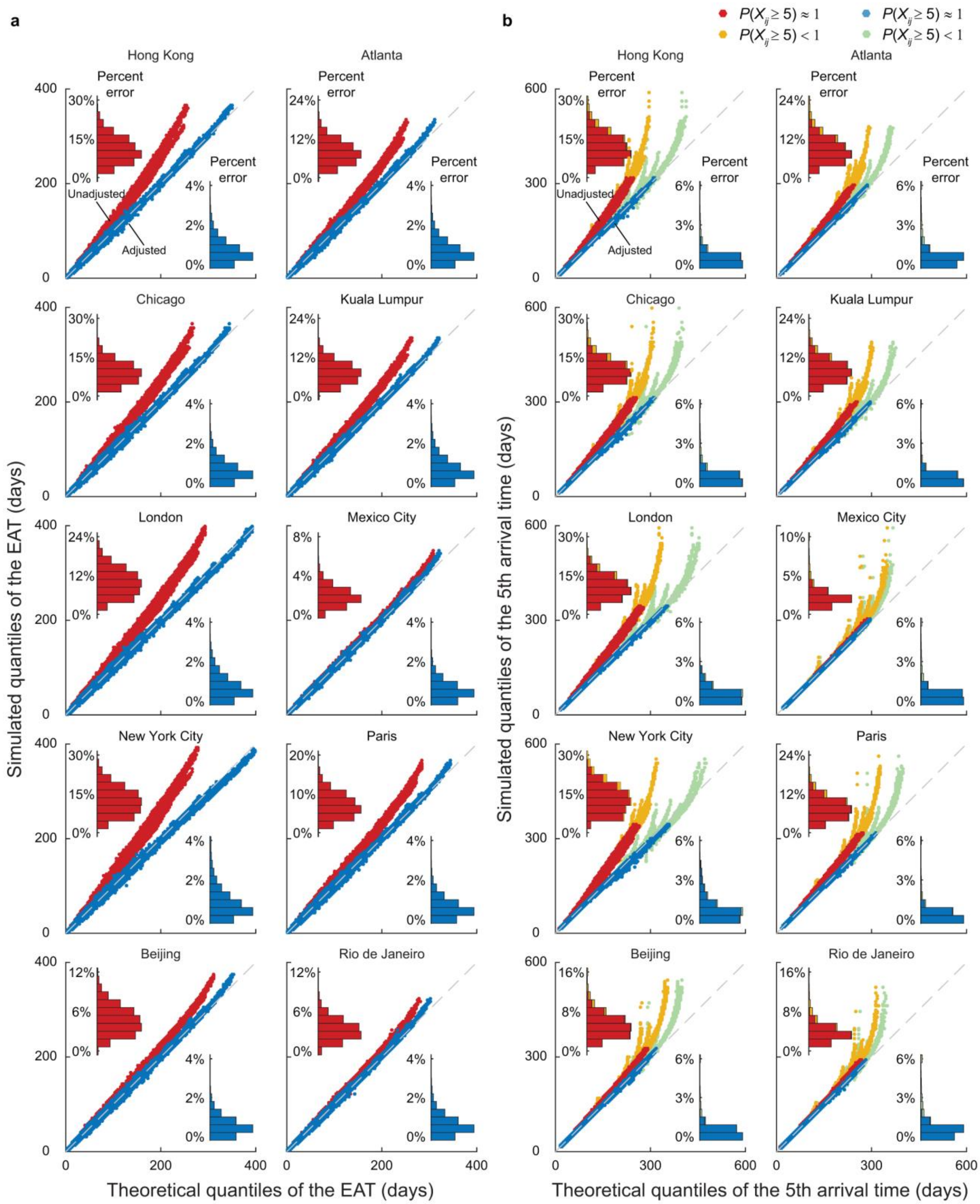

Supplementary Figure 3. Hub-effect adjustments for $D_{i,1}$ populations. a, Analogous to Fig. 2d with different major hubs in the WAN as the epidemic origin. **b**, Same as panel **a** but for the 5th arrival time T_{ij}^{5}.

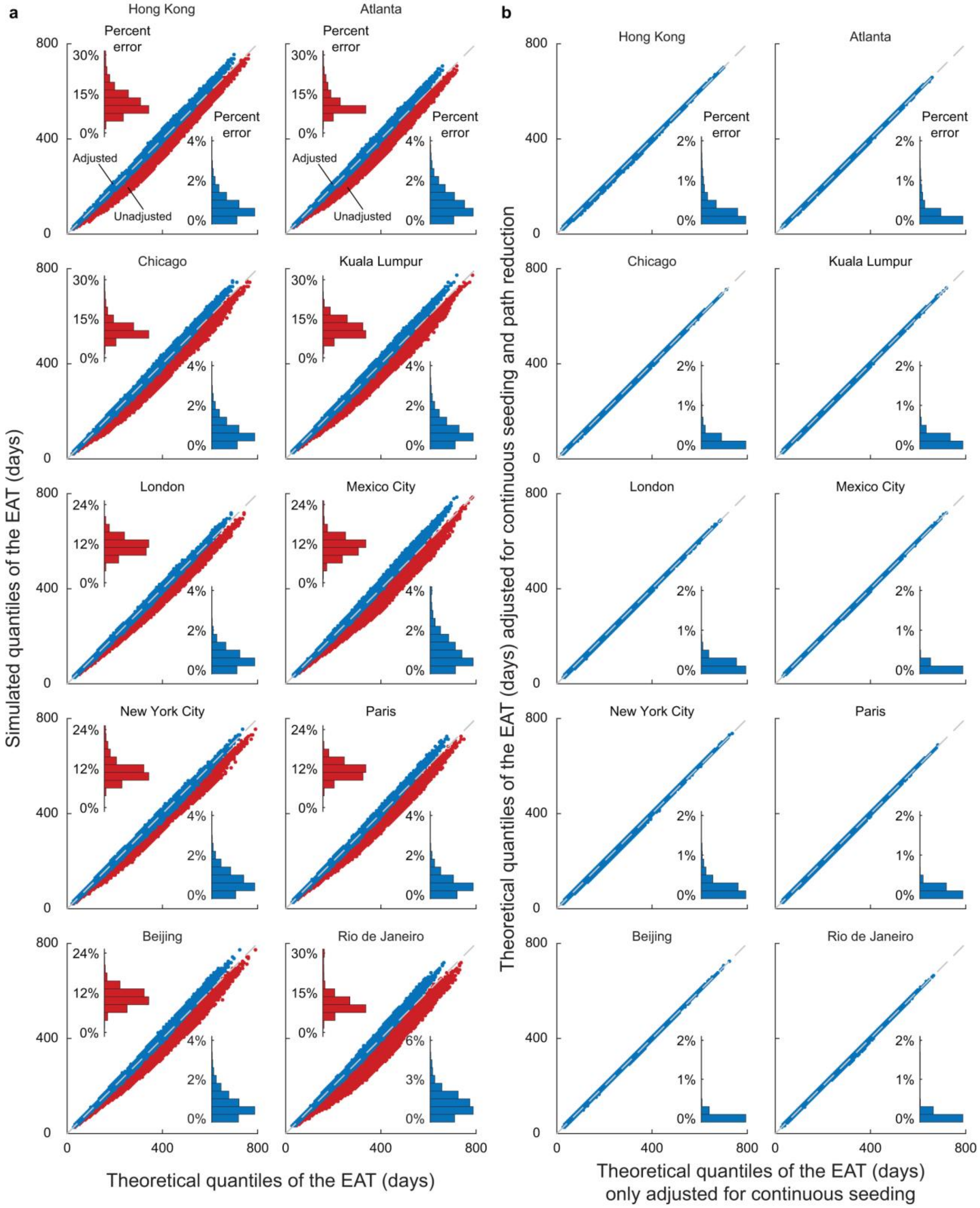

Supplementary Figure 4. Analogous to Fig. 2e for $D_{i,2}$ populations with different major hubs in the WAN as the epidemic origin. a, Q-Q plots for the analytical and simulated quantiles of EATs before (red) and after (blue) adjusting for continuous seeding. **b**, Q-Q plots for

the analytical quantiles of EATs with and without path reduction after adjusting for continuous seeding.

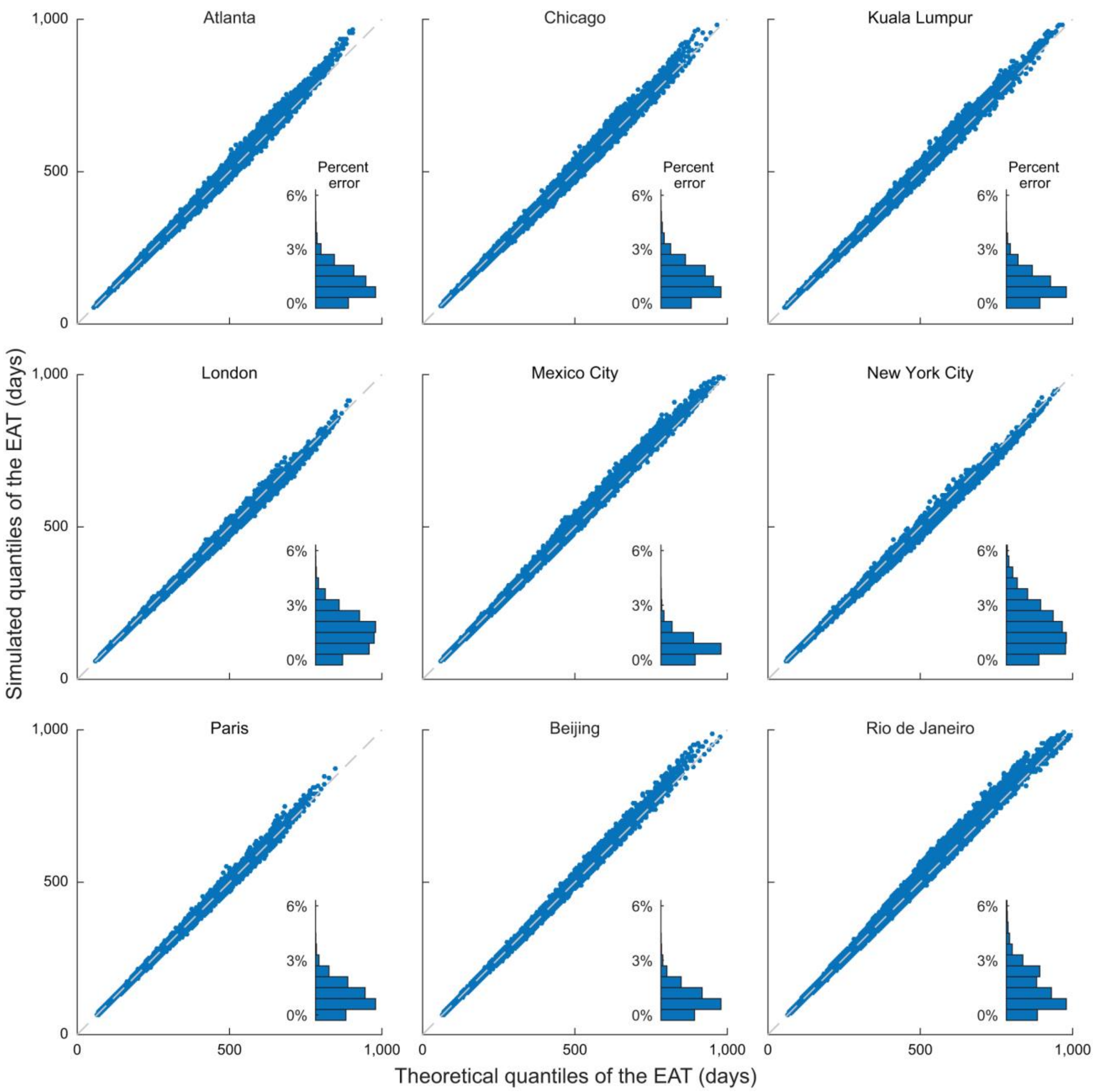

Supplementary Figure 5. Analogous to Fig. 2f for $D_{i,3}$ and $D_{i,4}$ (and $D_{i,5}$ if any) populations with different major hubs in the WAN as the epidemic origin.

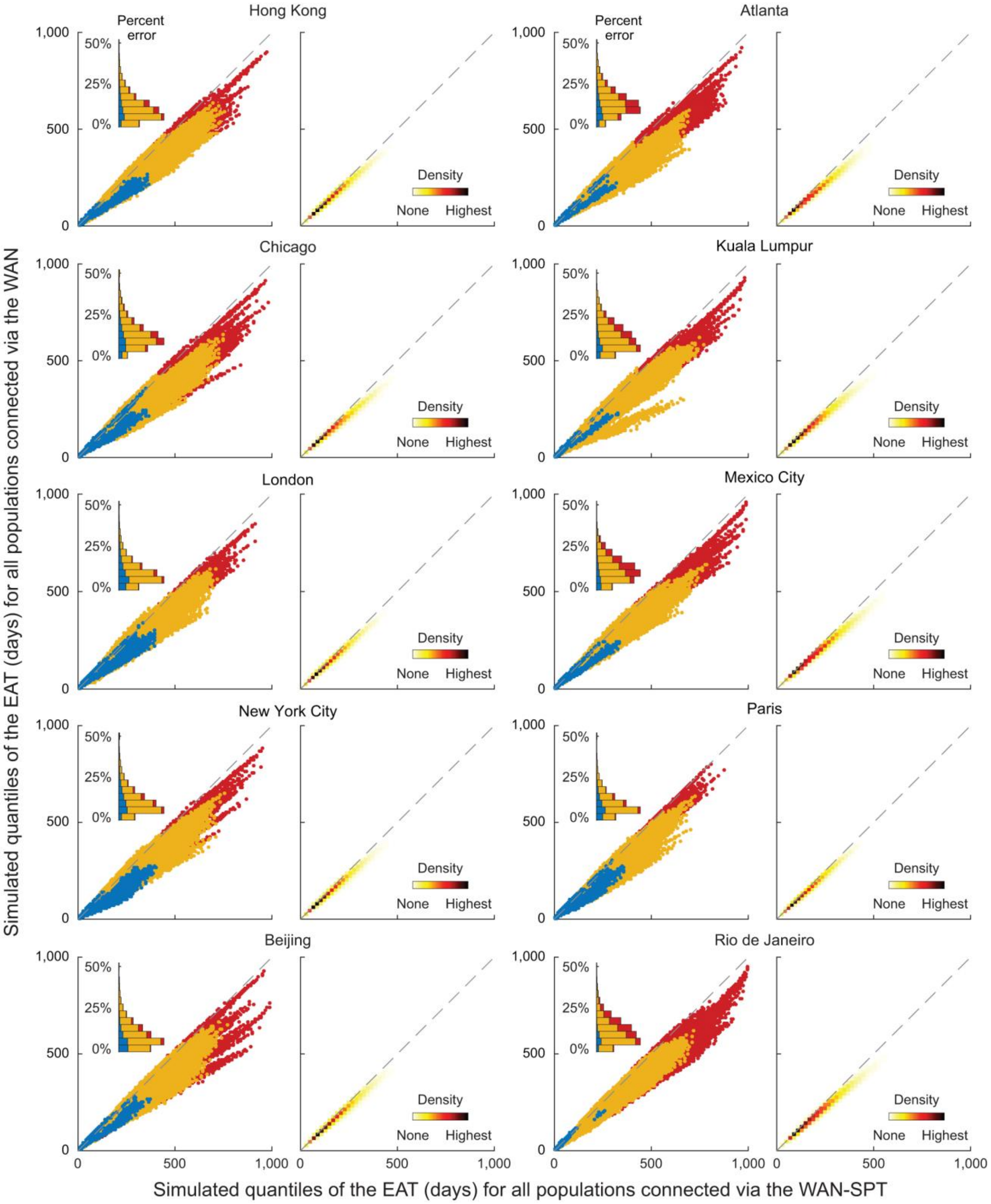

Supplementary Figure 6. Q-Q plots for the simulated EATs in the WAN-SPT and WAN and the corresponding density plots with different major hubs in the WAN as the epidemic origin.

a

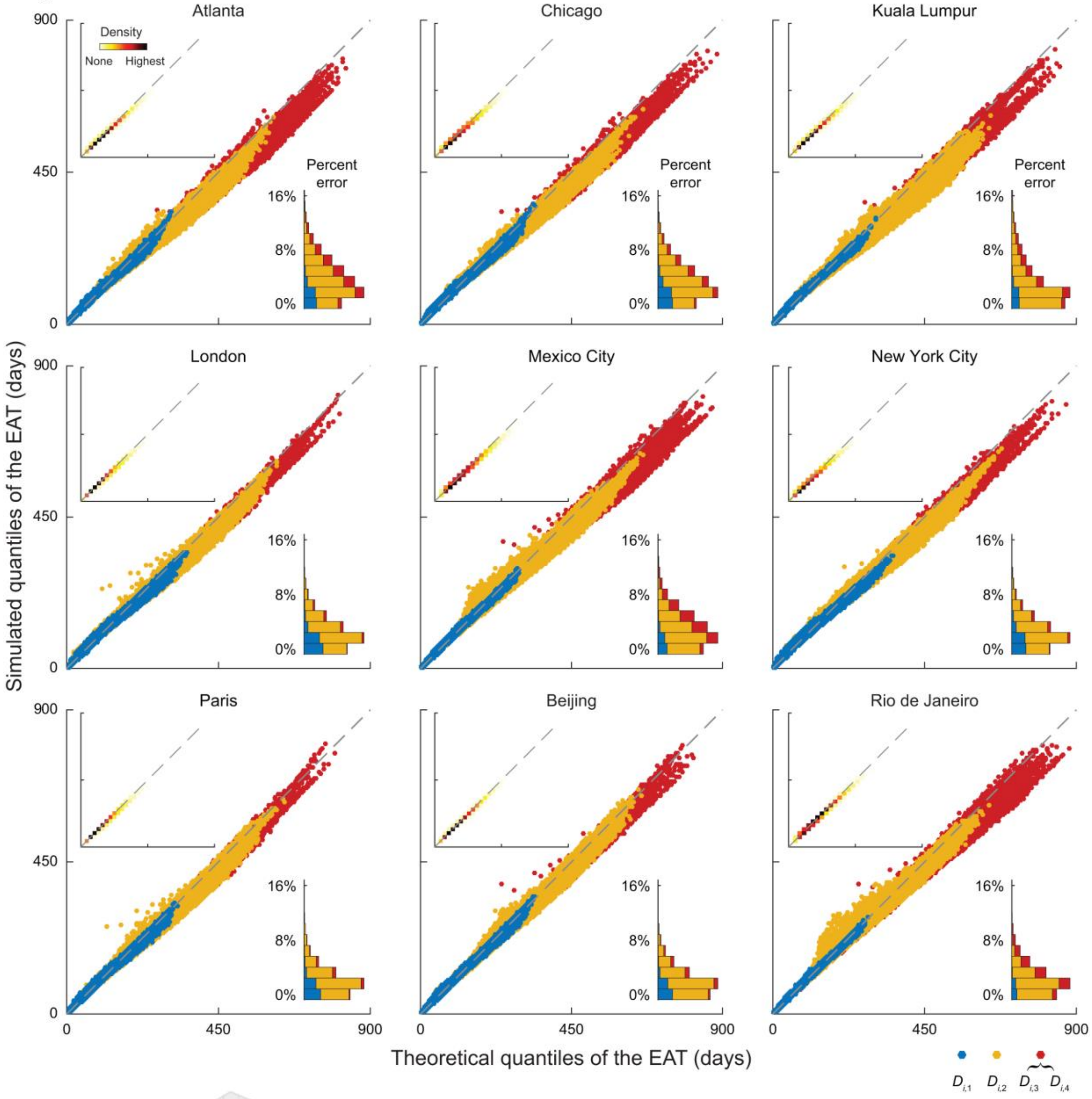

b

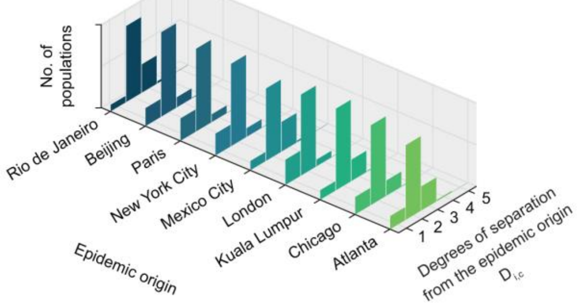

Epidemic origin	No. of populations				
	$D_{i,1}$	$D_{i,2}$	$D_{i,3}$	$D_{i,4}$	$D_{i,5}$
Atlanta	263	1528	490	25	2
Chicago	354	1634	309	11	n/a
Kuala Lumpur	190	1672	434	12	n/a
London	513	1660	132	3	n/a
Mexico City	185	1483	605	33	2
New York City	444	1687	174	3	n/a
Paris	455	1694	156	3	n/a
Beijing	366	1730	204	8	n/a
Rio de Janeiro	130	1557	578	41	2

Supplementary Figure 7. Analogous to Fig. 3 with other major hubs in the WAN as the epidemic origin.

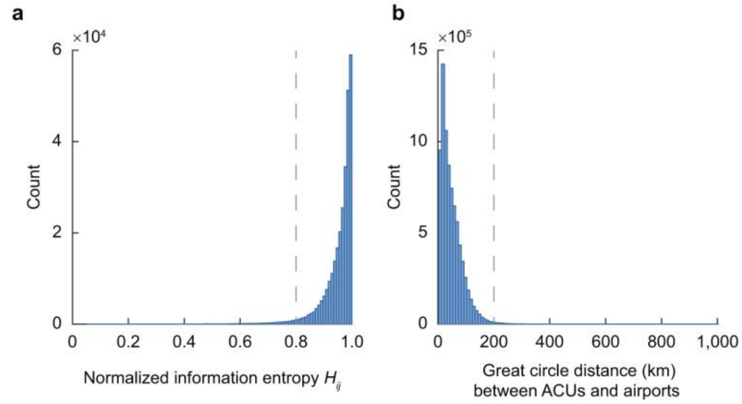

c

Summary statistics for the WAN after excluding weak flight routes

Cutoff*	No. of airports	No. of direct connections	Global traffic preseved (%)
0	2,726	276,665	96.4
1,000	2,702	111,830	94.6
2,000	2,664	80,922	93.1
3,000	2,594	66,327	92.0
4,000	2,528	57,388	90.8
5,000	2,471	51,208	89.8

* Flight routes with annual air bookings below this cutoff are excluded

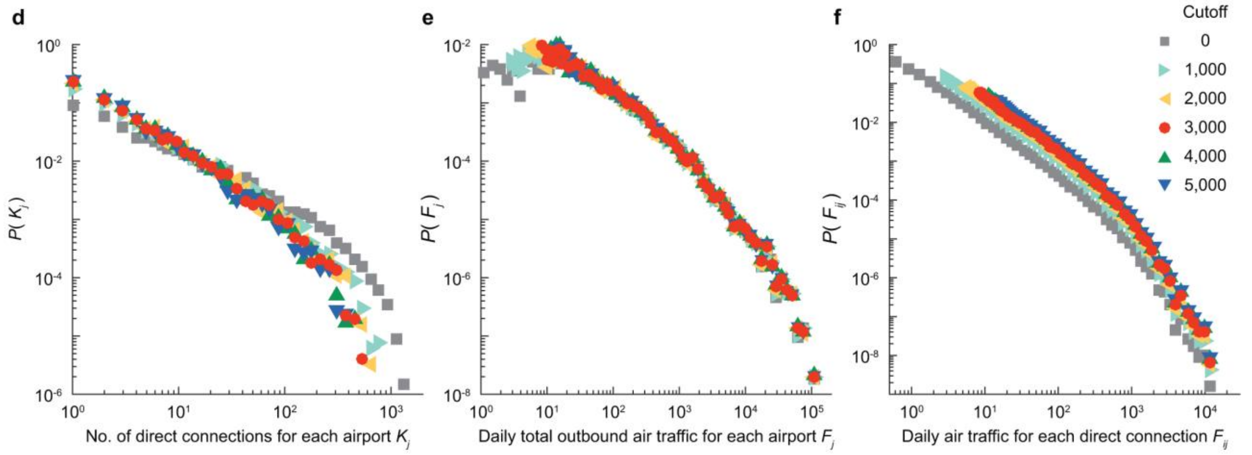

Supplementary Figure 8. Building the global epidemic simulator. a, Histogram of the seasonality measure H_{ij} (based on normalized information entropy) calculated with the raw data in the OAG flight booking dataset. **b**, Distribution of great circle distance for all combinations of ACUs in the original GPWv4 dataset and airports in the OAG dataset after flight routes with H_{ij} < 0.8 have been excluded. **c**, Summary network statistics with different cutoff for eliminating flight routes with weak traffic. **d–f**, Distributions of key network statistics with different cutoff for eliminating flight routes with weak traffic.

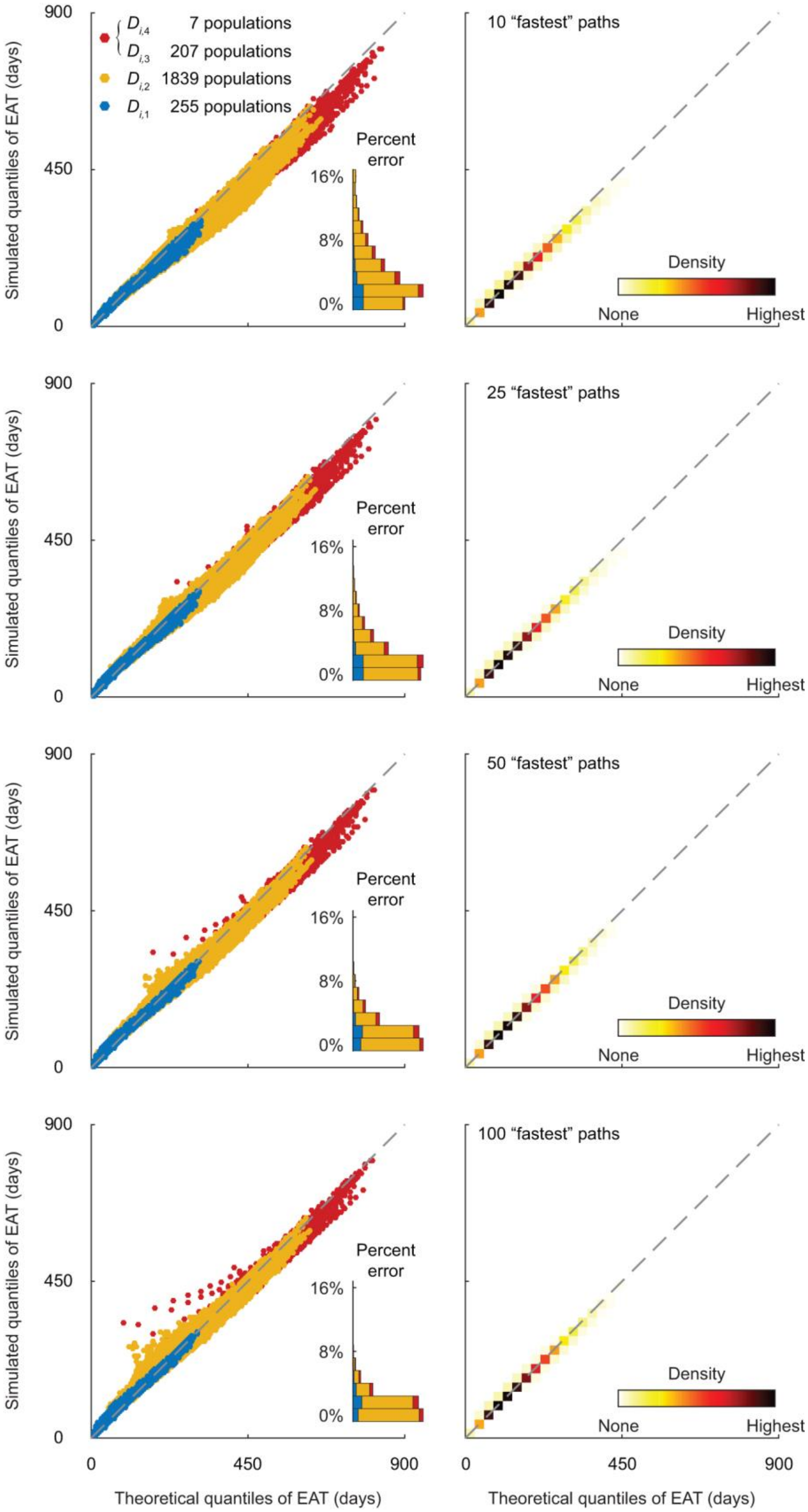
$D_{i,4}$ 7 populations
$D_{i,3}$ 207 populations
$D_{i,2}$ 1839 populations
$D_{i,1}$ 255 populations
Percent error
16%
8%
0%
10 "fastest" paths
25 "fastest" paths
50 "fastest" paths
100 "fastest" paths
Density
None
Highest
900
450
0
Simulated quantiles of EAT (days)
Theoretical quantiles of EAT (days)

Supplementary Figure 9. Superposition of paths in the WAN. Analogous to Fig. 3 with Hong Kong as the epidemic origin, this figure shows the effect of increasing the number of "fastest" paths for superposition on estimating the epidemic arrival times in the WAN. From top to bottom, the epidemic arrival time for each population in the WAN is computed with the superposition of the 10, 25, 50, and 100 “fastest” paths, respectively.

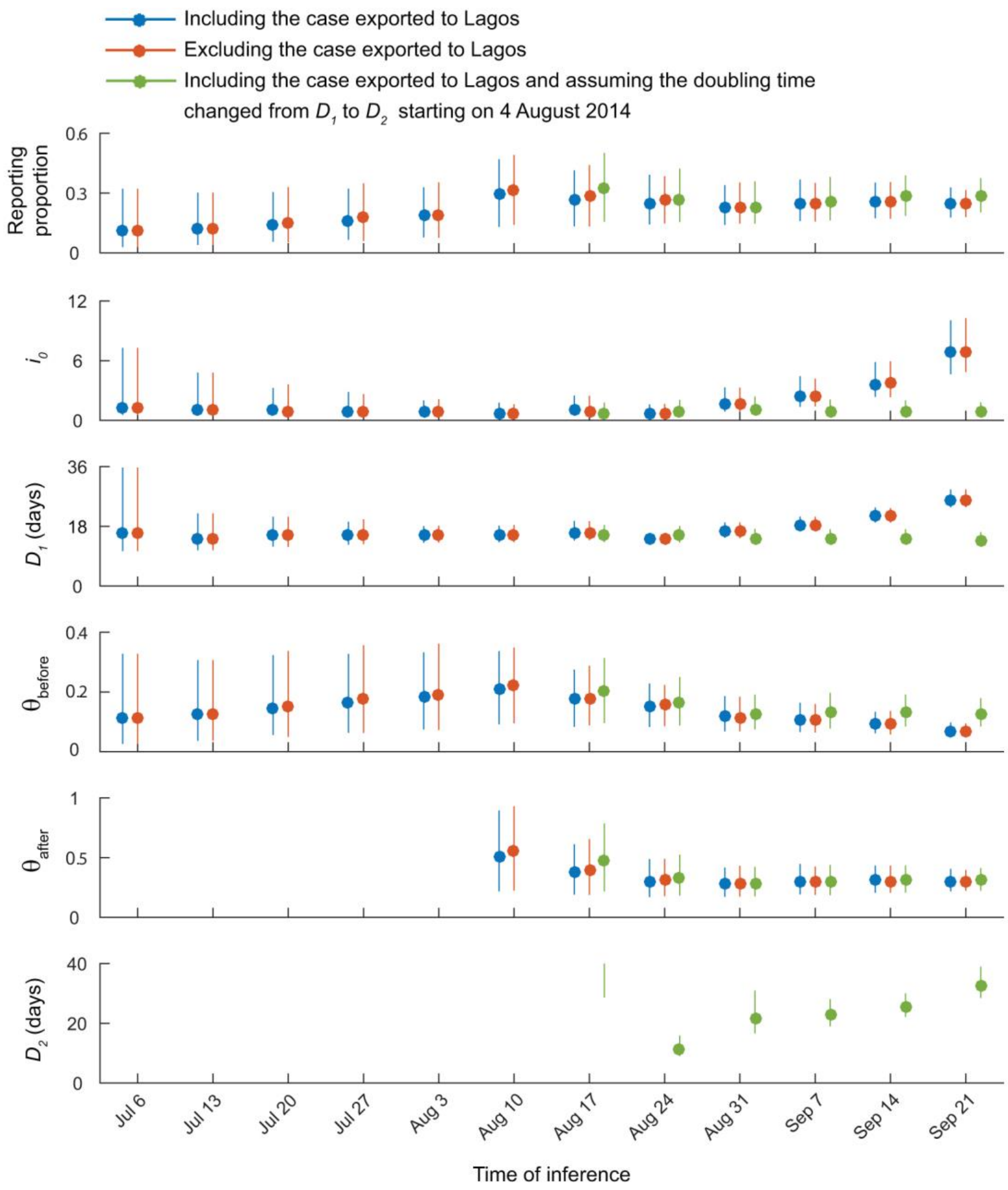

Supplementary Figure 10. Parameter estimates in the case study of the 2014 West African Ebola epidemic in Montserrado and Margibi, Liberia. Circles and bars indicate the medians and 95% credible intervals of the posterior distributions. Under the assumption that the doubling time changed from D_1 to D_2 starting on 4 August 2014, D_2 is statistically non-identifiable or unreliable until the week of 18-24 August 2014.

Supplementary Table 1. Raw data used in the case study of the 2009 influenza A/H1N1 pandemic in greater Mexico City.

Country*	Onset of symptoms†	Flight arrival†	Confirmation†	Air traffic during March-April 2009‡
US	28-Mar-09	n/a	21-Apr-09	545,368
Canada	11-Apr-09	8-Apr-09	23-Apr-09	52,702
El Salvador	n/a	19-Apr-09	3-May-09	15,090
UK	24-Apr-09	21-Apr-09	27-Apr-09	8,970
Spain	25-Apr-09	22-Apr-09	27-Apr-09	52,390
Cuba	n/a	25-Apr-09	13-May-09	23,100
Costa Rica	25-Apr-09	25-Apr-09	2-May-09	16,950
Netherlands	n/a	27-Apr-09	30-Apr-09	16,680
Germany	28-Apr-09	n/a	29-Apr-05	21,350
France	n/a	n/a	1-May-09	39,690
Guatemala	1-May-09	n/a	5-May-09	36,758
Colombia	n/a	n/a	3-May-09	24,535

* The 12 countries directly seeded by Mexico during 2009 influenza A/H1N1 pandemic [1].

† The epidemic arrival times for these 12 countries are based on the Supplementary Table 5 in Balcan et al. [1]. Given the first confirmed case imported in each country, its epidemic arrival time (EAT) is assumed to be (i) the onset date of symptoms or the date of flight arrival (whichever was earlier); or (ii) left-censored at the date of confirmation if neither the onset date nor the flight arrival date was reported.

‡ Number of air passengers departing from airports in greater Mexico City during March-April 2009, based on the Supplementary Table S3 in Fraser et al. [2].

Supplementary Table 2. Raw data used in the case study of the 2014 West African Ebola epidemic in Montserrado and Margibi, Liberia.

Month	No. of monthly total outbound international flight bookings*	Total outbound mobility rate in each month†
May	12,146	2.142×10^{-4}
Jun	12,760	2.326×10^{-4}
Jul	13,017	2.296×10^{-4}
Aug	12,417	2.190×10^{-4}
Sep	5,939	1.082×10^{-4}

* Monthly international air traffic departing from airports in Montserrado and Margibi, Liberia, between May and September 2014 is obtained from the OAG database. Flights to Guinea and Sierra Leone are excluded from analysis.

† The total population size of Montserrado and Margibi in Liberia is 1,828,846 in year 2015 according to the GPWv4 database.

Supplementary References

1 Balcan, D. *et al.* Seasonal transmission potential and activity peaks of the new influenza A(H1N1): a Monte Carlo likelihood analysis based on human mobility. *BMC Med.* **7**, 45, doi:10.1186/1741-7015-7-45 (2009).

2 Fraser, C. *et al.* Pandemic potential of a strain of Influenza A (H1N1): Early findings. *Science* **324**, 1557-1561, doi:10.1126/science.1176062 (2009).
